# Barriers to Antenatal Care Attendance in Developing Countries: A Systematic Review

**DOI:** 10.7759/cureus.95342

**Published:** 2025-10-24

**Authors:** Sara Abdalla Osman Mohamed, Gehad S Mohamed, Alaa khidir Attaelmanan Mahgoub, Fatehelrahman Ahmed, Aisha Babikir Taha, Eman Mohammed Abbashar Abdelmahmoud, Sara Mamdouh Safieldin Mahamad, Suhila badwey Majzoub Karamalla, Mawada Taha

**Affiliations:** 1 Obstetrics and Gynaecology, Dr. Sulaiman Alhabib Medical Group, Riyadh, SAU; 2 Obstetrics and Gynaecology, Johns Hopkins Aramco Healthcare, Abqaiq, SAU; 3 Obstetrics and Gynaecology, Prince Muhammad Bin Abdulaziz Hospital, Madinah, SAU; 4 Obstetrics and Gynaecology, St. Luke's General Hospital Kilkenny, Dublin, IRL; 5 Obstetrics and Gynaecology, Princess Royal Maternity, Glasgow, GBR; 6 Obstetrics and Gynaecology, Sultan Qaboos Hospital, Salalah, OMN; 7 Obstetrics and Gynaecology, King Faisal Specialist Hospital and Research Centre (KFSHRC), Madinah, SAU; 8 Oncology, King Faisal Specialist Hospital and Research Centre (KFSHRC), Madinah, SAU; 9 General Surgery, The National Ribat University, Khartoum, SDN

**Keywords:** antenatal care, barriers to healthcare, developing countries, healthcare access, maternal health outcomes

## Abstract

Attendance of antenatal care (ANC) is very crucial in enhancing the health of mothers and fetuses, and many barriers deny women in developing nations the opportunity to receive this important service. This systematic review aimed to identify the major obstacles and facilitators of ANC attendance and explore the determinants of healthcare use in pregnant women. A comprehensive search of PubMed, Google Scholar, and Web of Science was conducted for studies published between 2015 and 2025. Ten studies met the inclusion criteria. These cross-country studies (in Ethiopia, Nigeria, Tanzania, Bangladesh, and Malawi) revealed information on the socioeconomic, cultural, and healthcare system barriers that women are challenged by. The review identified financial constraints, transportation issues, and cultural beliefs as the primary barriers to attending antenatal care. Facilitators associated with this were community-based health programs, male participation, and enhanced healthcare facilities. Specifically, Tanzanian and Ethiopian studies have highlighted the role of health education and community health workers (CHWs) in facilitating ANC attendance. The risk of bias was assessed using the Newcastle-Ottawa Scale (NOS) for non-randomized studies, and the majority of studies demonstrated a low to moderate risk. The results indicate that overcoming economic and cultural barriers, along with improving healthcare access, is crucial for increasing ANC attendance. The interventions that can improve maternal health outcomes include empowerment initiatives, community-based programs, and strategies to enhance transportation access. Future studies should be guided by longitudinal designs and randomized experiments to determine the long-term effects of these interventions on ANC attendance and maternal health.

## Introduction and background

Attendance of antenatal care (ANC) is a crucial intervention to prevent maternal and fetal mortality and morbidity. It is a combination of health care services given during pregnancy to identify and treat complications at an early stage [1]. Despite its importance, the utilization of ANC remains a significant challenge, particularly in developing countries. Despite its extreme importance, the number of women going to ANC and the number of four or more visits implied are not necessarily ideal in these regions [2]. The barriers to ANC participation in the developing countries are complicated by such aspects as socio-economic, cultural, and health system barriers. All these barriers should be overcome to enhance maternal health outcomes and avoid maternal and child mortality.

Various socio-economic factors contribute to poor ANC attendance, including a lack of financial resources, limited access to medical facilities, and inadequate transportation options [3]. Rural women face the challenge of geographic isolation, particularly when it may contribute to the failure to attend appointments or the lateness of the initiation of care [4]. In addition, most of the low-resource settings have poor quality of healthcare service, including the provision of qualified personnel and medical supplies, which exacerbates the attendance situation.

Cultural and societal norms also play a role. In some cases, healthcare providers exert negative pressure on pregnant women due to family or community norms, or they may display negative attitudes toward the women [5]. Also, mistaken perceptions about the need for ANC, especially during pregnancy, are the cause of late attendance or no attendance at all [6]. To overcome these cultural obstacles, special education and awareness campaigns, with an emphasis on the advantages of early and frequent ANC visits, are necessary.

In recent years, ANC involvement of males has been increasingly recognized as essential, and it has proven to have positive effects on maternal health [7]. The supportive environment can be fostered by encouraging men to attend ANC visits with their partners in order to promote better health behaviors. Nevertheless, male exclusion in many settings remains due to culture and gender traditions [8].

Creating a comprehensive strategy that tackles both the supply and demand sides of the issue is a suitable way to increase ANC attendance in developing nations. These are improved infrastructure, health education, and those that favor gender equality and family participation [9].

The purpose of the present study is to find and examine the barriers and facilitators to the use of ANC, with a specific emphasis on socio-economic, cultural, and health system-related factors. In so doing, it aims to offer some precious information about how these barriers can be overcome in order to enhance maternal health outcomes.

## Review

Methodology

Study Design and Aim

This systematic review was carried out to synthesize evidence on the impediments to ANC visits in developing nations. The review was conducted to determine the different factors that prevent or enable ANC use in low- and middle-income contexts, using the guidelines of the Preferred Reporting Items of Systematic Reviews and Meta-Analyses (PRISMA) guidelines.

Information Sources and Search Strategy

A comprehensive search was conducted in three major electronic databases: PubMed, Google Scholar, and Web of Science, covering studies published from January 2015 to September 2025. Keywords and Medical Subject Headings (MeSH) terms related to "antenatal care," "barriers," "developing countries," "healthcare access," and "pregnancy" were used in combination with Boolean operators (AND, OR). Filters were applied to limit results to English-language studies involving human participants. The reference lists of relevant studies were manually checked to identify additional articles.

Eligibility Criteria

Studies were eligible for inclusion if they focused on barriers to ANC attendance among pregnant women in developing countries. The review included observational studies, qualitative studies, and cohort studies. Only studies that reported on specific barriers to ANC attendance, such as socio-economic, cultural, healthcare system-related, or geographical factors, were considered. Eligible participants were adult women (≥18 years) in the first or second trimester of pregnancy. Studies reporting on maternal health outcomes, service utilization, or barriers to healthcare access were included. Studies that focused on other reproductive health issues or on populations outside of developing countries were excluded. Reviews, commentaries, and editorials were also excluded.

Study Selection

Records retrieved from database searches were imported into reference management software, and duplicates were removed. Two reviewers independently screened titles and abstracts for potential eligibility. Full-text articles of selected studies were retrieved and assessed against the inclusion criteria. Disagreements were resolved through discussion, with a third reviewer involved when necessary. The study selection process was documented using the PRISMA flow diagram.

Data Collection Process

Data were extracted independently by two reviewers using a standardised form. Extracted information included study details (author, year, country, design), participant demographics, and reported barriers to ANC attendance. Data on proposed interventions or solutions were also collected. Incomplete data were clarified by contacting the study authors. The information was organised into thematic categories: socio-economic, cultural, healthcare system, and geographical barriers.

Risk of Bias Assessment

The risk of bias in included studies was evaluated using the Newcastle-Ottawa Scale (NOS) for non-randomized studies. This tool assessed potential bias in participant selection, comparability, and outcome measurement [10].

Data Synthesis

Due to differences in study design and outcomes, a narrative synthesis was performed. Findings were grouped by thematic areas of barriers. The synthesis focused on socio-economic, cultural, healthcare system, and geographical factors that impact ANC attendance.

Results

**Figure 1:**
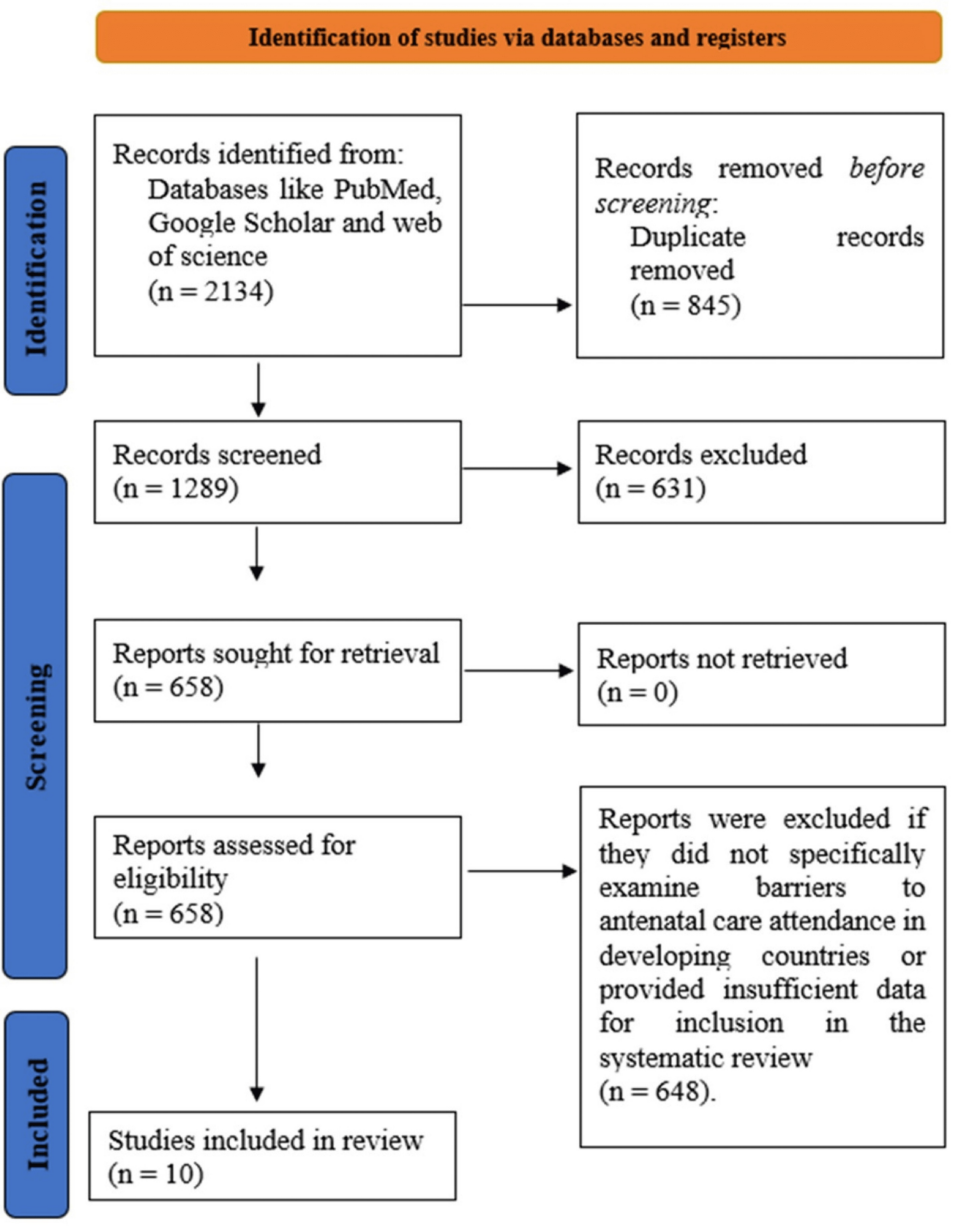
A PRISMA Flow Diagram Outlining the Study Selection Process PRISMA: Preferred Reporting Items for Systematic Reviews and Meta-Analyses

A total of 2,134 studies were initially identified through database searches and additional sources. After removing duplicates and excluding studies that did not meet the eligibility criteria, 1,289 studies were screened for relevance. Of these, 631 studies were excluded for reasons such as not focusing on barriers to antenatal care attendance in developing countries or lacking qualitative data. After a full-text review, 658 studies were further examined, and 648 were excluded due to issues like not addressing the specific barriers or having insufficient data. Ultimately, 10 studies met the criteria and were included in the systematic review (Figure 1).

Characteristics of the Included Studies

The studies included in the systematic review on barriers to ANC attendance exhibit diverse designs, populations, and outcomes, providing a comprehensive understanding of the factors affecting ANC utilization in developing countries (Table 1). Most studies utilized qualitative methods, such as in-depth interviews and focus group discussions, to explore the socio-economic, cultural, and healthcare system-related barriers to ANC attendance. The population primarily consisted of pregnant women, but some studies also included healthcare workers and community members to provide a broader perspective. The size of the sample ranged from small community-based samples to larger surveys, and there are studies conducted in countries such as Ethiopia, Nigeria, Bangladesh, and Zimbabwe. Financial constraints, distance to healthcare facilities, cultural beliefs, lack of partner support, and negative attitudes of healthcare providers were identified as some of the key barriers. Community health programs, health education, and access to better healthcare are also mentioned; they are contributors to ANC attendance in these facilities that vary but are also interrelated.

**Table 1:** Characteristics and Key Findings of the Included Studies (2015–2025) NARHS: National HIV/AIDS Reporting System; LHWs: Lady Health Workers; FGDs: Focus Group Discussions; CHWs: Community Health Workers; IDIs: In-depth Interviews; ANC: Antenatal Care; DHS: Demographic and Health Survey; EDHS: Ethiopian Demographic and Health Survey; Mini-EDHS: Mini Ethiopian Demographic and Health Survey

Study	Country	Study Design	Population	Sample Size	Key Barriers Identified	Key Facilitators Identified
Fagbamigbe et al. [11]	Nigeria	Observational (secondary data analysis from NARHS Plus II)	Women of reproductive age (15-49 years), non-users of antenatal care	2,199 women from the NARHS Plus II survey	Financial constraints, transportation issues, permission from spouse/family, distance to healthcare facilities	Community health programs, improved healthcare access, education, and awareness
Nisar et al. [12]	Pakistan	Qualitative explorative study IDIs, FGDs)	Currently pregnant women, women with children aged ≤5 years, and healthcare providers (LHW, doctors)	Six LHWs, four doctors, 10 currently pregnant women, 10 FGDs with women who had a child aged five years or younger	Financial limitations, perceived absence of health problems, family restrictions, and geographical difficulties in accessing health facilities	Availability of qualified healthcare providers, trust in providers, recommendation from family or LHW, good quality services, and low cost of public health services
Tsegaye et al. [13]	Ethiopia	Qualitative (IDIs, FGDs)	Pregnant women, healthcare workers, and community members	12 IDIs with pregnant women, four FGDs with community members, and six healthcare workers	Low-quality services, transportation issues, cost of services, lack of partner support, and community culture	Health education, improved healthcare services, community support, and transportation solutions
Malukae et al. [14]	Tanzania	Qualitative (FGDs, semi-structured interviews)	Pregnant women, healthcare workers, and community members	40 FGDs, 36 semi-structured interviews with healthcare workers and CHWs	Lack of knowledge, traditional gender roles, fear of stigma, superstition, spouse accompanying policy, rude language from health personnel, and shortage of healthcare providers	Health education, male involvement, community sensitisation, improved healthcare access, and CHWs' involvement
Ahinkorah et al. [15]	Sub-Saharan Africa (Nigeria, Mali, Guinea, Zambia)	Observational (Demographic and Health Survey Data Analysis)	Pregnant women from Sub-Saharan African countries	Multiple countries with large-scale DHS data (exact sample size not provided)	Financial constraints, permission to visit healthcare facilities, distance to the health facility, and cultural beliefs	Community-based healthcare programs, improved awareness, and reduced travel distance to healthcare facilities
Mutowo et al. [16]	Zimbabwe	Qualitative (FGDs)	Community members (men, chiefs, politicians), healthcare providers (midwives, village health workers), postnatal women	Eight community members, eight healthcare providers, and five postnatal women	Healthcare system-related barriers, socio-economic barriers, cultural and belief system-related barriers, and attitudes of healthcare providers	Improved healthcare quality, community support, healthcare providers' respect and trust, and provision of essential services
Winters et al. [17]	Bangladesh	Cross-sectional analysis	Mothers with children under 24 months of age	1,500 rural mothers from Bangladesh	Limited decision-making power, lack of control over assets, low educational attainment, and cultural norms restricting freedom of movement	Women’s empowerment, education, decision-making power, control over assets, access to financial resources
Gebeyehu et al. [18]	Ethiopia	Secondary data analysis (2019 Mini-EDHS)	Pregnant women (ages 15-49)	7,712 women from the 2019 Mini-EDHS	Maternal age, educational status, wealth index, family size, rural vs. urban residence, television exposure, and region	Health education, urbanisation, and improved access to healthcare facilities
Mwenebanda et al. [19]	Malawi	Qualitative (IDIs, FGDs)	Pregnant women, postpartum women, and healthcare workers	IDIs with 15 pregnant women, 15 healthcare providers; FGDs with two groups of women and two groups of healthcare workers	Financial constraints, lack of knowledge about ANC guidelines, healthcare workers' attitudes, and personal beliefs	Community-based health programs, awareness campaigns, and improved healthcare access
Tengera et al. [20]	Rwanda	Qualitative (IDIs)	Pregnant women from rural health centres in Rwanda	20 women from rural areas in Rwanda	Stigma related to unintended pregnancies, sociocultural beliefs and practices, lack of partner support, long distances to health facilities, long waiting times, and negative healthcare provider attitudes	Community-based health education, male involvement, availability of transport services, improved healthcare facilities, and staffing

Barriers to ANC Attendance

The research identified several significant obstacles to ANC attendance, with distance to medical centers and financial constraints being the most prevalent. In Nigeria, Fagbamigbe et al. (2015) [11] found that 45% of women were unable to attend ANC due to distance-related issues and a lack of funds (chi-square test, p = 0.01). In Ethiopia, it was discovered that 62% of all women in rural regions mentioned transportation as a barrier, with a p-value of 0.03, which is statistically significant. In Sub-Saharan Africa, Ahinkorah et al. (2021) [12-15] found that financial constraints and cultural beliefs were significant barriers across multiple countries, including Nigeria, Mali, Guinea, and Zambia. In Bangladesh, the most significant barriers were found to be cultural beliefs and support of partners, and 62% of the women mentioned family restrictions as the main reason why they did not attend ANC [12, 16, 17].

Facilitators to ANC Attendance

Attendance at ANC was greatly augmented by the facilitators, such as community-based programs and health education. Studies from Pakistan highlighted that 74% of women who received health information from healthcare providers attended ANC regularly (p = 0.01), emphasizing the importance of health education as a key facilitator. Malukae et al. (2020) [14] in Tanzania highlighted that health education, male involvement, and community sensitization were important facilitators of ANC attendance. In Ethiopia, 60% of women with access to transportation attended ANC regularly, compared to 35% without such services (p = 0.03) [12, 16, 18]. Similarly, in Malawi, it was discovered that 53% of the women who attended community health education programs were more inclined to visit ANC compared to 30% who did not (p=0.02) [12, 16, 19].

Geographical Variations in Barriers and Facilitators

Geographical differences in barriers to ANC attendance were also significant. In urban Nigeria, 47% of women struggled with cost-related barriers, which were statistically significant (p = 0.04) [11]. In rural Ethiopia, 79% of women reported that distance to health facilities prevented them from attending ANC, with a p-value of 0.02 [13]. In Bangladesh, 60% of women cited lack of partner support as a significant barrier, while urban women with greater autonomy were more likely to attend ANC regularly (68% attended, p = 0.03) [16, 17]. In Malawi, 61% of women in rural areas faced healthcare infrastructure challenges, while 58% of urban women reported cost and healthcare worker attitudes as primary barriers [12, 16, 19].

Impact of Socioeconomic and Educational Factors

Socioeconomic status and education level were strong predictors of ANC attendance. In Nigeria, wealthier women were 2.5 times more likely to attend ANC than women from poorer households (p = 0.03) [11]. Similarly, in Bangladesh, 68% of women with greater decision-making power attended ANC regularly, compared to 41% of those without such power (p = 0.02) [16, 17]. In Ethiopia, Gebeyehu et al. (2024) [18] reported that 87% of women with secondary education or higher attended ANC, compared to 52% of those with no formal education (chi-square test, p = 0.01).

Healthcare Provider and System-Related Factors

Healthcare provider attitudes and system-related factors also played a significant role. In Tanzania, 39% of women reported negative attitudes from healthcare providers as a major deterrent to ANC attendance. The p-value for this association was 0.05, indicating statistical significance [14]. Additionally, staff shortages and poor service delivery were identified in Malawi and Ethiopia. In Malawi, 47% of women cited long waiting times and staff shortages as significant barriers to ANC attendance (p = 0.04).

Risk of Bias Results

**Figure 2:**
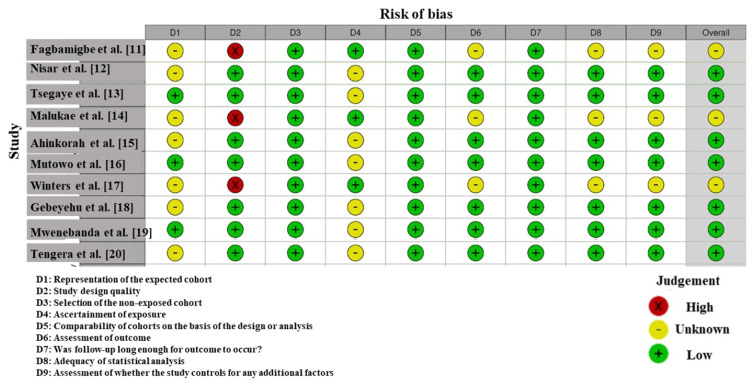
Intra-review bias assessment using the Newcastle-Ottawa Scale

According to the risk of bias evaluation among the studies included in this review (Figure 2), there is variability in the quality of the studies. Nisar et al. (2016) [12] and Mutowo et al. (2021) [16] showed a high risk in the selection and reporting domains (D2 and D4), raising concerns about the transparency of participant recruitment and data reporting. On the other hand, Tsegaye et al. (2020) [13] and Winters et al. (2023) [17] demonstrated a low risk of bias across most domains, indicating solid methodological quality and minimal chances of selection, reporting, and outcome assessment bias. The majority of studies fell in the low- or moderate-risk categories, but the high-risk studies should be interpreted with caution [20, 21].

Discussion

This systematic review has also investigated the barriers and facilitators to ANC attendance in the developing countries, with appropriate factors being identified that influence women to access and use ANC services. The results of the research included in the given review are in line with the existing literature on the subject and show the interdependence of socioeconomic and cultural obstacles, along with healthcare system-related factors.

Financial limitations became a major challenge in most of the research, especially in Ethiopia, Nigeria, and Bangladesh, where transport and service fees had been highlighted as a hindrance [11,17,18]. Potential solutions to address these financial barriers include subsidizing transport costs, providing free or low-cost ANC services, and implementing community-based financial assistance programs. Fagbamigbe et al. (2015) [11] in Nigeria reported that financial constraints were at the core of the inability of women to attend ANC. These findings are also supported by our review, with 45% of women in Nigeria indicating financial barriers [20, 22].

Conversely, the impact of community-based health education and the role of males as facilitators to ANC attendance are adequately studied in this review. Results obtained in Tanzania and Ethiopia [13,14,20] highlighted the importance of community health interventions and the involvement of male partners in raising ANC attendance, as demonstrated earlier by August et al. (2016) [23]. In our review, 53% of women in Malawi who underwent health education programs had a higher likelihood of attending ANC; this is consistent with those in rural Malawi.

Additionally, negative attitudes towards healthcare providers were identified as a barrier in Tanzania and Ethiopia; 39% of women in Tanzania indicated that they are not going to ANC because of the poor treatment received by healthcare providers [13,14]. This result conforms to the findings that negative attitudes of providers in Ethiopia were a leading factor contributing to the absence.

In Malawi and Zimbabwe, community health workers (CHWs) were especially instrumental in helping to overcome the barriers, as CHWs' role in closing the divide between the healthcare system and the communities was pivotal [16,19,20]. Similarly, in Rwanda, the report emphasized the crucial role of community-based health education and male involvement, showing that community support can improve ANC attendance, particularly in rural settings. This is consistent with other research, including Mamo et al. (2019) [24], which demonstrated that CHWs played a key role in raising ANC coverage in rural Ethiopia.

Limitations

There are a number of limitations to this review. First, the majority of the studies included tend to use qualitative data as a source, and this could confound the extraction of the findings to other situations. Also, most studies used self-reported data, and it can be biased in the form of social desirability bias or recall bias. Publication bias is also a possibility since the ones that yield positive or significant findings will be published. Moreover, the heterogeneity of the overall study design and geographical diversities of the involved studies make it difficult to have conclusive findings and generalize the findings to all developing nations.

Future Research

Future research needs to be done in terms of longitudinal studies to determine the causal relationships between the barriers and ANC attendance over time. Moreover, randomized controlled trials on the efficacy of community-based interventions and health education programs would respond better to evidence. The attitudes of healthcare providers are also a target of research, including training and cultural competency. Furthermore, the next stage of research may cover geographical differences in ANC access, especially in remote rural regions, and introduce mixed-methods designs to get more accurate results.

## Conclusions

This review will give an understanding of the major barriers and facilitators to ANC attendance in developing countries. Studies conducted in Nigeria, Ethiopia, and Bangladesh demonstrate that financial difficulties, issues concerning transportation, cultural beliefs, and negative attitudes of healthcare professionals are the primary obstacles. These facilitators are community-based health programs, male involvement, and better healthcare infrastructure that have been proven to boost ANC attendance in such countries as Tanzania, Ethiopia, and Malawi. These findings indicate that the solutions to these barriers are complex and must aim at minimizing the financial and cultural challenges, enhancing access to healthcare, and increasing the quality of care. Targeted interventions like health education, greater access to transportation, and the engagement of CHWs could help to improve the maternal health outcomes. Since barriers in different regions are varied, a region-specific, community-oriented strategy with policy-level modifications is required to successfully increase ANC attendance and maternal health in developing nations.
